# 4354 The Impact of the 2014 Kidney Allocation System on Waitlisting Rates at the Dialysis Facility Level

**DOI:** 10.1017/cts.2020.437

**Published:** 2020-07-29

**Authors:** Taylor Andrew Melanson, Jennifer Gander, Rachel Patzer

**Affiliations:** 1 Emory University; 2 Kaiser Permanente Georgia Regional; 3 Emory University School of Medicine

## Abstract

OBJECTIVES/GOALS: The new Kidney Allocation System (KAS) was implemented in 2014 and it is not fully understood how its changes to patient incentives may have impacted dialysis facility waitlisting rates. We examine differences in facility performance and how such differences may have been impacted by this policy change. METHODS/STUDY POPULATION: We used Dialysis Facility Report data from 2011 to 2017 to study waitlisting rates at 3,392 dialysis facilities in the US, using waitlisting counts in the numerator, and the total number of ESRD patients in a facility as the denominator. We examined changes in waitlisting rates over by year at the facility, regional, and national level, and report national trends in waitlisting pre- and post-KAS. Facilities were stratified based on waitlisting rate in 2011 and then we examined whether each facility moved into a higher or lower quartile or stayed in the same quartile in 2017. RESULTS/ANTICIPATED RESULTS: Among n = 3,392 dialysis facilities, the average change in dialysis facility waitlisting rates from 2011 to 2017 was −4.74 percentage points (range -54.4% to 42.3%). Average change in dialysis facility waitlisting rates from 2011 to 2014 was −0.57 percentage points while the average change in dialysis facility waitlisting rates from 2014 to 2017 was −4.17 percentage points. Half of facilities in the 2011 lowest quartile remained in the lowest quartile in 2017; 45% of facilities in the top 2011 quartile dropped into a lower quartile. The middle 2 quartiles were fairly evenly split between worsening, improving, and not changing. DISCUSSION/SIGNIFICANCE OF IMPACT: Average waitlisting rates have declined since KAS implementation. Many facilities switched quartiles from 2011-17 suggesting that facility waitlisting rates are unstable over time. The decline in waitlisting rates post-KAS suggests that new allocation rules may be discouraging patients and/or providers from getting ESRD patients waitlisted.

